# The British Laryngological, Rhinological, and Otological Society

**Published:** 1898-02-19

**Authors:** 


### THE BRITISH LARYNGOLOGICAL, RHINOLOGICAL, AND OTOLOGICAL SOCIETY.

At the last meeting, Mr. Ballance read a paper on Some Lessons in the Diagnosis of Intracranial Complications in Otitis Media, gleaned from twelve fatal cases. Among other things, he recommended that in operating in cases of thrombosis, the jugular vein should be fastened to the skin incision. In discussing this paper, Mr. Milligan (Manchester) said that he had used antistreptococcic serum in a case of septicæmia following chronic suppurative middle-ear disease, but it had not proved of the slightest value. In Manchester they had had an unusual experience of cerebral abscesses following influenza, in several of which the abscess had been located in the frontal lobe. Mr. George Reid read notes of a case of Traumatic Rupture of the Tympanic Membrane in a child aged nine years, caused by a fall on the point of the chin. A linear perforation was found in the posterior segment, running in the direction of the fibres, with raw edges from which blood was oozing. There was slight temporary deafness, but no other injury of the head whatever. Dr. Wyatt Wingrave showed a man, aged 48, suffering from malignant ulceration of the tonsil, extending to the faucial pillar and tongue, which had commenced as a small sore on the tonsil, and was at first thought to be of a syphilitic nature, and had, in fact, once entirely disappeared under the use of iodide of potassium. Mr. Marsh agreed with Mr. Wingrave that epithelioma frequently supervened upon tertiary syphilitic ulceration in the buccal cavity. Mr. Furniss Potter showed a case of malignant disease infiltrating the left side of the larynx, and involving the arytenoid region, the ventricular band, and the ary-epiglottic fold. The only symptoms complained of were hoarseness for nine weeks previously, with slight pain shooting into the left ear occasionally.

